# Spatiotemporal Expression of MANF in the Developing Rat Brain

**DOI:** 10.1371/journal.pone.0090433

**Published:** 2014-02-28

**Authors:** Haiping Wang, Zunji Ke, Alexander Alimov, Mei Xu, Jacqueline A. Frank, Shengyun Fang, Jia Luo

**Affiliations:** 1 Department of Molecular and Biochemical Pharmacology, University of Kentucky College of Medicine, Lexington, Kentucky, United States of America; 2 Institute for Nutritional Sciences, SIBS, CAS, Shanghai, China; 3 Center for Biomedical Engineering and Technology, Department of Physiology, University of Maryland School of Medicine, Baltimore, Maryland, United States of America; University of Kentucky, United States of America

## Abstract

Mesencephalic astrocyte-derived neurotrophic factor (MANF) is an evolutionarily conserved neurotrophic factor which exhibited neuroprotective properties. Recent studies suggested that MANF may play a role in the neural development of *Drosophila* and zebra fishes. In this study, we investigated the spatiotemporal expression of MANF in the brain of postnatal and adult rats. MANF expression appeared wide spread and mainly localized in neurons. In the cerebral cortex, neurons in layer IV and VI displayed particularly strong MANF immunoreactivity. In the hippocampus, intensive MANF expression was observed throughout the subfields of Cornu Amonis (CA1, CA2, and CA3) and the granular layer of the dentate gyrus (DG). In the substantia nigra, high MANF expression was shown in the substantia nigra pars compacta (SNpc). In the thalamus, the anterodorsal thalamic nucleus (ADTN) exhibited the highest MANF immunoreactivity. In the hypothalamus, intensive MANF immunoreactivity was shown in the supraoptic nucleus (SON) and tuberomammillary nucleus (TMN). In the cerebellum, MANF was localized in the external germinal layer (EGL), Purkinje cell layer (PCL), internal granule layer (IGL) and the deep cerebellar nuclei (DCN). We examined the developmental expression of MANF on postnatal day (PD) 3, 5, 7, 9, 15, 21, 30 and adulthood. In general, the levels of MANF were high in the early PDs (PD3 and PD5), and declined gradually as the brain matured; MANF expression in the adult brain was the lowest among all time points examined. However, in some structures, such as PCL, IGL, SON, TMN and locus coeruleus (LC), high expression of MANF sustained throughout the postnatal period and persisted into adulthood. Our results indicated that MANF was developmentally regulated and may play a role in the maturation of the central nervous system (CNS).

## Introduction

Mesencephalic astroctye-derived neurotrophic factor (MANF) is a 20 kDa secreted protein and is first identified as arginine-rich, mutated in early stage of tumor (ARMET). It was recognized as a protein with a high mutation rate in various tumors [1], [2]. It is now known that MANF together with cerebral dopamine neurotrophic factor (CDNF) belongs to a *novel* evolutionary conserved protein family which show neurotrophic and neuroprotective activities [3]. MANF promotes the survival of rat embryonic nigral dopaminergic neurons, but not serotonergic or GABAergic neurons *in vitro* [4]. Intrastriatal injection of recombinant MANF protects dopaminergic neurons against 6-hydroxydopamine (6-OHDA)-induced neurodegeneration [5]. MANF transcripts and protein levels are up-regulated after ischemic and epileptic insults in the cerebral cortex, suggesting that it may have neuroprotective effects against neurotoxins and cerebral ischemia [5]–[8]. We have recently showed that MANF was induced in response to ethanol neurotoxicity in the developing brain [9]. MANF is an endoplasmic reticulum (ER) stress responsive protein and shown to suppress ER stress-induced cell death [8], [10]–[14].

MANF mRNA was detected in both neural and non-neural tissues. In mouse brains, relative high *manf* mRNA levels are detected in the cerebral cortex, hippocampus and cerebellum; it is also detected in the liver, heart, lung, kidney and testis [6]. Recently, MANF has been found to regulate the development of dopaminergic neurons in *Drosophila* and zebra fishes [15]–[17]. The MANF homologous gene in *Drosophila, DmMANF* is required at the end of *Drosophila* embryogenesis for the maturation of the nervous system. *DmMANF* maternal and zygotic null mutants caused a total loss of dopaminergic neurites and drastic reduction in dopamine levels followed by degeneration of axonal bundles and subsequent cell death [15], [17]. Similarly, knockdown of MANF expression decreased the dopamine levels and the expression of tyrosine hydroxylase gene transcripts in larval zebra fishes [18]. These findings suggest that MANF may play a role in neural development. To determine the potential involvement of MANF in the development of central nervous system (CNS), we investigated the spatiotemporal expression of MANF in the brain of postnatal and adult rats. We demonstrated that MANF was developmentally regulated; high levels of MANF were present in early postnatal days and its expression declined as the brain matured.

## Materials and Methods

### Materials

Rabbit anti-MANF antibody was purchased from Abcam (Cambridge, MA). Recombinant MANF protein was expressed and purified as previously described [10]. Mouse anti-neuronal nuclei (NeuN) antibody was obtained from Millipore Corporate (Billerica, MA). Mouse anti-glial fibrillary acidic protein (GFAP) and calbindin antibodies were obtained from Sigma Chemical Co. (St. Louis, MO). Mouse anti-tyrosine hydroxylase antibody was purchased from BD biotechnology (San Diego, CA). Biotin-conjugated anti-mouse and anti-rabbit secondary antibodies and the ABC kit were obtained from Vector (Burlingame, CA). Alexa-488 conjugated anti-rabbit and Alexa-594 conjugated anti-mouse antibodies were obtained from Life Technologies (Grand Island, NY). Mouse anti-actin, HRP-conjugated anti-rabbit and anti-mouse secondary antibodies were purchased from GE Healthcare Life Sciences (Piscataway, NJ). Ketamine/xylazine was obtained from Butler Schein Animal Health (Dublin, OH). Other chemicals and reagents were purchased either from Sigma Chemical or Life Technologies.

### Animals

Pregnant Sprague-Dawley rats were obtained from Harlan Laboratories (Indianapolis, IN) and maintained in the Division of Laboratory Animal Resources of the University of Kentucky Medical Center. All procedures were performed in accordance with the guidelines set by the National Institutes of Health (NIH) *Guide for the Care and Use of Laboratory Animals* and were approved by the Institutional Animal Care and Use Committee at the University of Kentucky.

### Preparation of Brain Tissues and Immunoblotting

Rat pups were anesthetized by an intraperitoneal injection of ketamine/xylazine (100 mg/kg/10 mg/kg) and cerebral cortices were dissected on postnatal day (PD) 1, PD3, PD5, PD7, PD9, PD15, PD21 and PD30. The brain tissues were immediately frozen in liquid nitrogen and then stored at −80°C. The protein was extracted and subjected to immunoblottting analysis as previously described [9]. Briefly, brain tissues were homogenized in an ice cold lysis buffer containing 50 mM Tris-HCl (pH 7.5), 150 mM NaCl, 1 mM EGTA, 1 mM PMSF, 0.5% NP-40, 0.25% SDS, 5 µg/ml leupeptin, and 5 µg/ml aprotinin. Homogenates were centrifuged at 20,000 g for 30 min at 4°C and the supernatant fraction was collected. After determining the protein concentration, equal amount of protein from each pup at the same time point was collected and pooled. Aliquots of the protein samples (30 µg) were separated on a SDS-polyacrylamide gel by electrophoresis. The separated proteins were transferred to nitrocellulose membranes. The membranes were blocked with either 5% BSA or 5% nonfat milk in 0.01 M PBS (pH 7.4) and 0.05% Tween-20 (TPBS) at room temperature for one hour. Subsequently, the membranes were probed with primary rabbit anti-MANF antibody overnight at 4°C. After three quick washes in TPBS, the membranes were incubated with a secondary antibody conjugated to horseradish peroxidase (GE Healthcare Life Sciences, Piscataway, NJ). The immune complexes were detected by the enhanced chemiluminescence substrate (PerkinElmer, Waltham, MA). In some cases, the blots were stripped and re-probed with either an anti-tubulin or an anti-actin antibody. The density of immunoblotting was quantified with the software of Quantity One (Bio-Rad Laboratories, Hercules, CA).

### Immunohistochemistry

The procedure for immunohistochemistry (IHC) has been previously described with some modifications [19]. Briefly, animals were deeply anesthetized with intraperitoneal injection of ketamine/xylazine and then intracardially perfused with PBS followed by 4% paraformaldehyde in PBS (pH 7.4). The brains were removed, post fixed in 4% paraformaldehyde for an additional 24 hours and then transferred to 30% sucrose in PBS. They were then frozen in OCT compound and sagittal sections at 40 µm were dissected on a freezing sliding microtome (Leica Microsystems, Wetzlar, Germany). Floating sections were incubated in 0.3% H_2_O_2_/30% methanol in PBS for 10 min. After washing with PBS, sections were mounted on slides and dried on a 50°C heat blocker. The slides were then blocked with 5% goat serum and 0.5% TritonX-100 in PBS for 1 hour at room temperature. After blocking, the slides were treated with a rabbit anti-MANF antibody (1∶6,000) overnight at 4°C. After washing with PBS, slides were incubated with biotin conjugated goat anti-rabbit secondary antibody (1∶1,000) for 1 hour at room temperature and followed by washing with PBS. Avidin-biotin-peroxidase complex was prepared according to the manufacturer’s instructions. The slides were incubated in the complex for 1 hour at room temperature. After rinsing, the slides were developed in 0.05% 3,3′-diaminobenzidine (DAB) (Sigma-Aldrich, Inc.) containing 0.003% H_2_O_2_ in PBS.

### Double-labeling Immunofluorescent Staining

The procedure for double-labeling immunofluorecent staining has been previously described with some modifications [19]. Briefly, rat pups of PD15 were anesthetized and perfused as described above. The brain sections were prepared at 15–20 µm thickness, mounted and dried on a 50°C heat blocker. After blocking with 5% goat serum and 0.5% TritonX-100 in PBS for 1 hour at room temperature, the slides were incubated with a rabbit anti-MANF antibody together with one of the antibodies for neural cell markers (GFAP, NeuN, calbindin or tyrosine hydroxylase) overnight at 4°C. After rinsing in PBS, the sections were incubated with Alexa Fluor488-conjugated anti-rabbit and Alexa Fluor 594-conjugated anti-mouse IgG in the dark at room temperature for 1 hour. The brain sections were examined and recorded by a fluorescent microscopy (IX81, Olympus).

### Statistical Analysis

Quantitative data were presented as the means ± SD. Statistical comparisons were analyzed using ANOVA followed by Dunnett’s test. The results with p<0.05 were considered statistically significant.

## Results

### MANF Expression in the Cerebral Cortex

**Figure 1:**
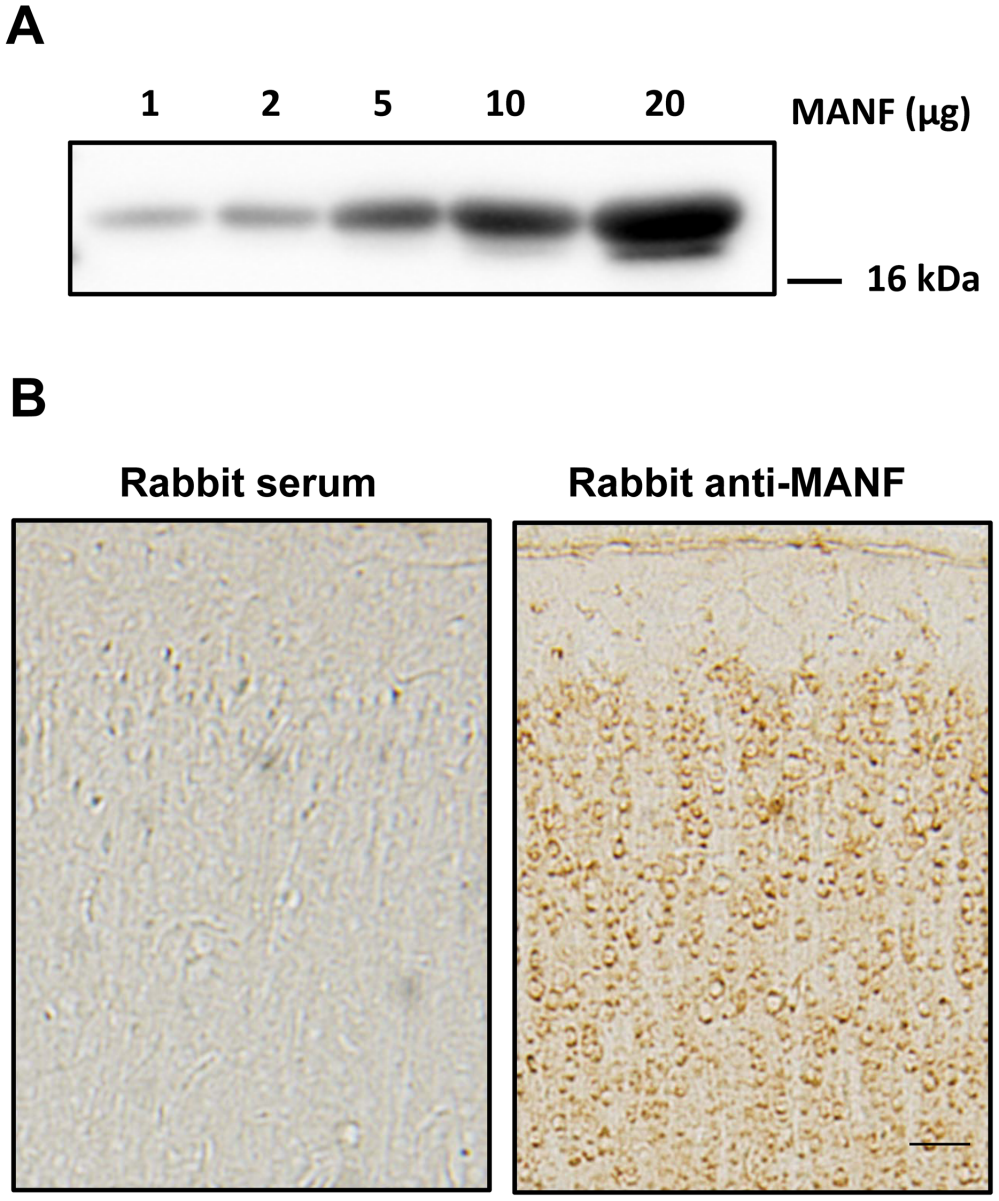
Detection of MANF. **A**: Different amount of human recombinant MANF (1–20 µg) were loaded on a SDS-polyacrylamide gel and subjected to immunoblotting analysis using a rabbit anti-MANF antibody. **B**: Rat pups of PD15 were sacrificed and perfused. The brain was dissected, sectioned and subjected to MANF immunohistochemistry (IHC) using a rabbit anti-MANF antibody (1∶6,000) as described in the Material and Methods. Rabbit serum (1∶6,000) was used as a control. Images were taken from the cerebral cortex. Scale bar = 100 µm.

To determine the specificity of anti-MANF antibody for immunoblotting and immunohistochemistry (IHC), we first used this antibody to perform an immunoblotting analysis on the recombinant MANF at various concentrations. We detected a single band at around 20 kDa and the intensity of signals increased as the concentrations of recombinant MANF increased (Fig. 1A). We further performed IHC on the cerebral cortex of rat pups of postnatal days (PD) 15 using either this anti-MANF antibody or normal rabbit serum. Positive signals were only detected for IHC using MANF antibody but not rabbit serum (Fig. 1B). These results indicated that this antibody specifically detected MANF.

**Figure 2:**
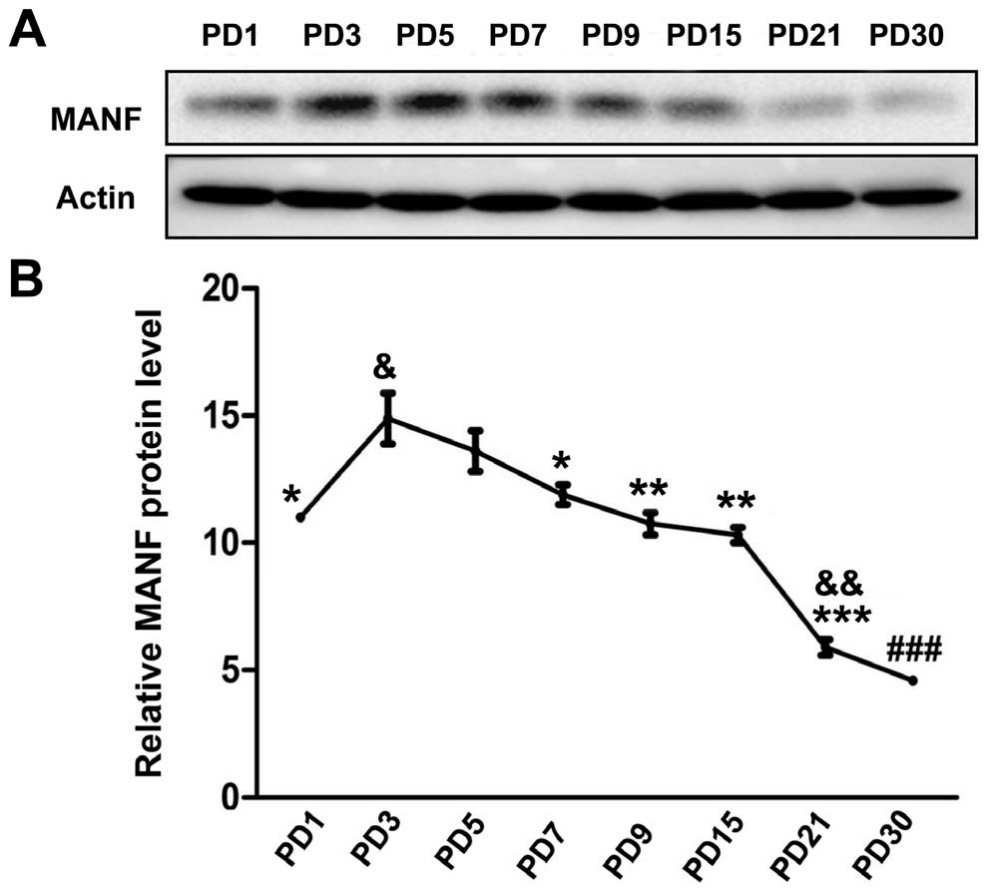
Study of developmental expression of MANF in the cerebral cortex by immunoblotting analysis. **A**: Rat pups were sacrificed at the indicated postnatal days (PD1-30). The cerebral cortex was dissected and homogenized. Equal amount of sample was pooled (3–5 pups per group) and subjected to immunoblotting analysis using an anti-MANF antibody described in Fig. 1. **B**: The relative expression of MANF was quantified by densitometric analysis. & p<0.05, && P<0.01 compared with PD1; *p<0.05, **P<0.01, ***P<0.001 compared with PD3, and ### P<0.001, compared with other groups.

**Figure 3:**
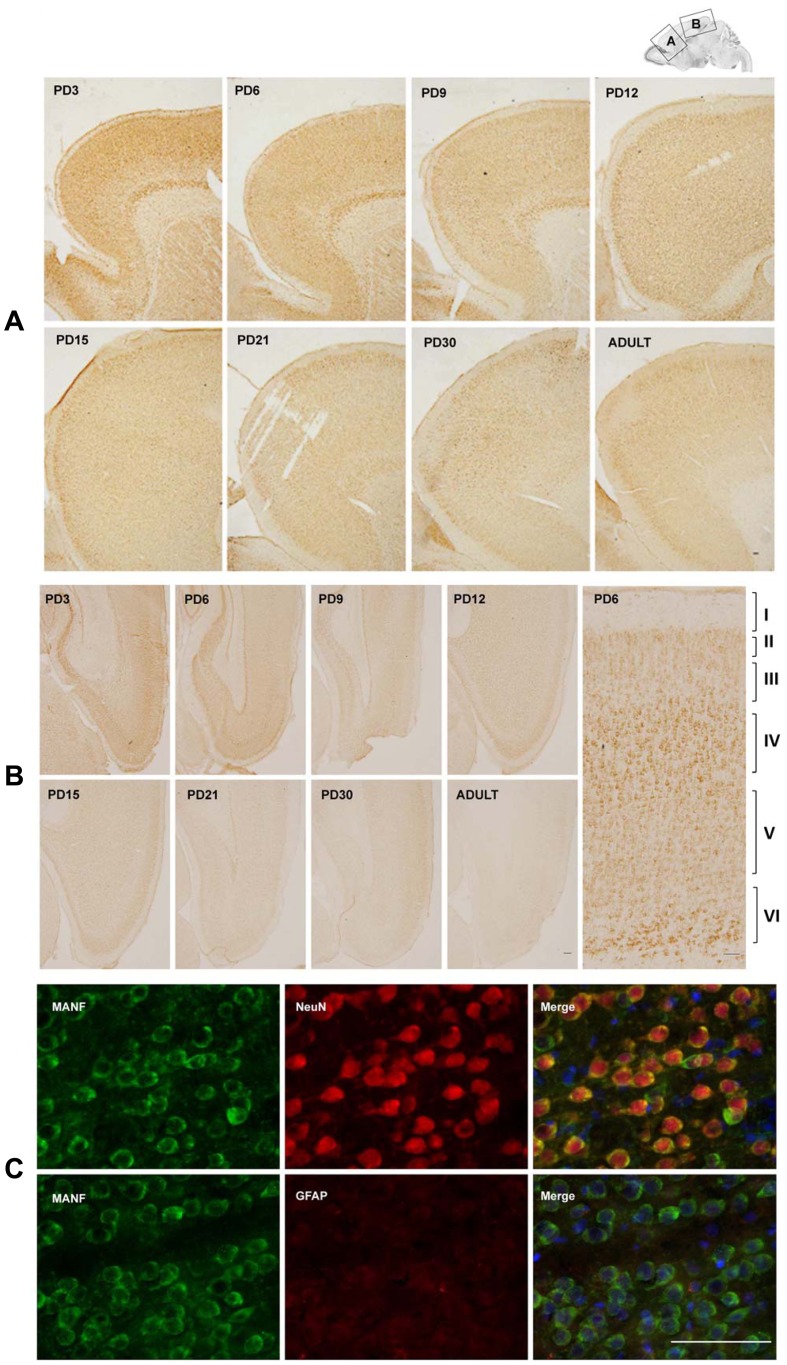
Study of developmental expression of MANF in the cerebral cortex by immunohistochemistry (IHC). **A and B**: MANF expression in the frontal, parietal and occipital cortex. IHC images were taken from indicated cortical areas shown above. A image of higher magnification showed layers in the cortical cortex (**B**). **C**: Double labeling immunofluorescent staining was performed to determine the localization of MANF (green). Neurons and astrocytes were identified by NeuN and GFAP (red), respectively. Images were generated from layer IV of the cerebral cortex of PD15 pups. Scale bar = 100 µm.

MANF is expressed in the cerebral cortex of mice [6]. We first examined the developmental expression of MANF in the cerebral cortex of rat pups by immunoblotting analysis (Fig. 2). As shown in Fig. 2, the expression levels of MANF were highest on PD3, gradually decreased as the brain matured, and were the lowest on PD30 (Fig. 2). We examined the distribution of MANF in the cerebral cortex by IHC. Strong MANF signal was detected in the frontal, parietal and occipital cortex on PD3 (Figs. 3A and B). Higher levels of MANF were found in layer IV and VI of the cortex in comparison to other layers (Fig. 3B). Consistent with the results obtained from immunoblotting analysis, the expression of MANF in the cortex decreased as the brain developed; among all time points examined, MANF expression was the lowest in the adult cerebral cortex (Figs. 3A and B). We used double-labeling immunofluorecent staining to determine the identity of cells expressing MANF. Neuronal-specific nuclear protein (NeuN) and glial fibrillary acidic protein (GFAP) were used as the markers for neurons and astrocytes, respectively. MANF was dominantly expressed in NeuN-positive cells (Fig. 3C). There was little MANF signal in glial GFAP-positive cells. The results indicated that MANF was mainly expressed in neurons.

### MANF Expression in the Substantia Nigra

**Figure 4:**
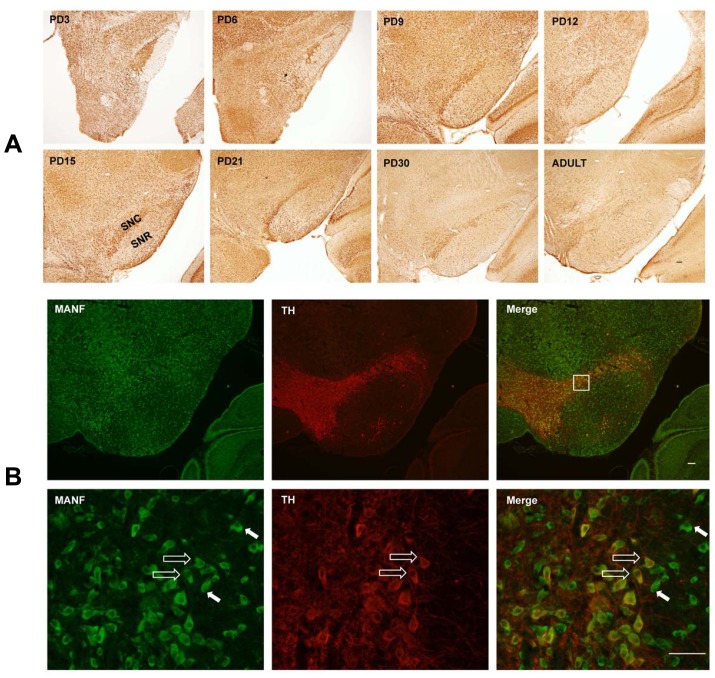
MANF expression in the substantial nigra. **A**: Rat pups were sacrificed at the indicated postnatal days. MANF IHC in substantial nigra was shown. SNC, Substantia Nigra pars Compacta; SNR, Substantia nigra pars reticulata. **B**: Double-labeling immunofluorescent staining was performed to determine the localization of MANF (green) in the SNC of PD15 pups. Dopaminergic neurons were indicated by tyrosine hydroxylase (TH) (red). The indicated square in the top panel is shown at a higher magnification in the bottom panel. TH- positive and negative cells were indicated by open arrows and solid arrows respectively. Scale bar = 100 µm.

MANF plays an important role in the maintenance and protection of mesencephalic dopaminergic neurons [4], [5], [11], [15]. We examined the expression of MANF in the developing substantia nigra and striatum. Strong MANF immunoreactivity was observed in the substantia nigra pars compacta (SNpc), and relatively weak expression was shown in substantia nigra pars reticulata (SNpr) (Fig. 4A). Double-labeling immunofluorescent analysis indicated that MANF was localized in both tyrosine hydroxylase (TH)-positive and negative cells in the SNpc, suggesting MANF was not specifically expressed in dopaminergic neurons (Fig. 4B). MANF expression was also detected in the caudate nucleus (CPu) of the striatum (data not shown), The temporal pattern of MANF expression in substantial nigra and CPu was similar to that of the cerebral cortex, that is, a higher expression was observed during the early postnatal days (PD3-6) and its expression decreased as the brain matured.

### MANF Expression in the Hippocampus, Thalamus and Hypothalamus

**Figure 5:**
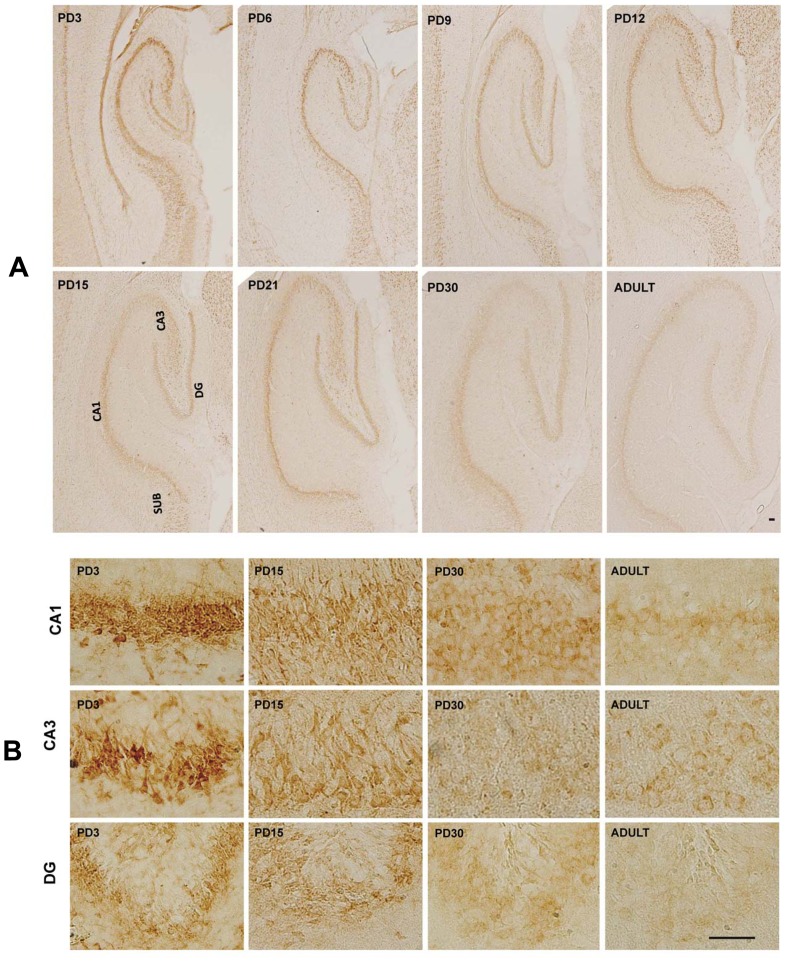
MANF expression in the hippocampus. **A**: Rat pups were sacrificed at the indicated postnatal days. MANF expression in the hippocampus was determined by IHC. CA1 and CA3, Cornu Ammonis 1 and 3 region of hippocampus; DG, dentate gyrus; SUB, subiculum. **B**: Image of higher magnification showed CA1, CA3 and DG of hippocampus. Scale bar = 100 µm.

High expression levels of MANF were observed in the hippocampus (Fig. 5). Intensive MANF staining was detected in CA1, CA2 and CA3 as well as in the granule cell layer (GCL) of dentate gyrus (DG) during the early postnatal days (PD3-6). MANF was expressed mainly in NeuN-positive cells (data not shown). The expression of MANF in the hippocampus decreased as the brain developed.

**Figure 6:**
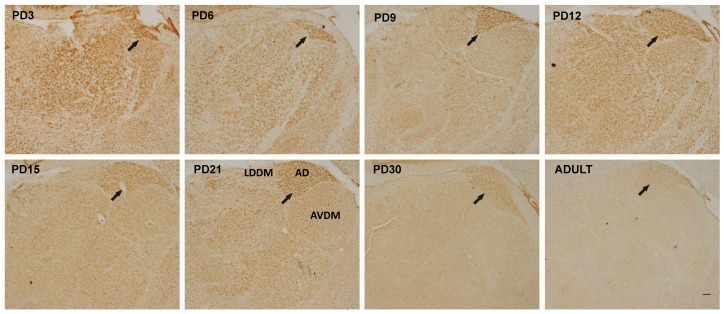
MANF expression in the thalamus. Rat pups were sacrificed at the indicated postnatal days. MANF expression in the hippocampus was determined by IHC. Arrows indicated the anterodorsal thalamic nucleus (ADTN). AVDM, anteroventral thalamic nucleus, dorsomedial part; LDDM, laterodorsal thalamic nucleus, dorsomedial part. Scale bar = 100 µm.

**Figure 7:**
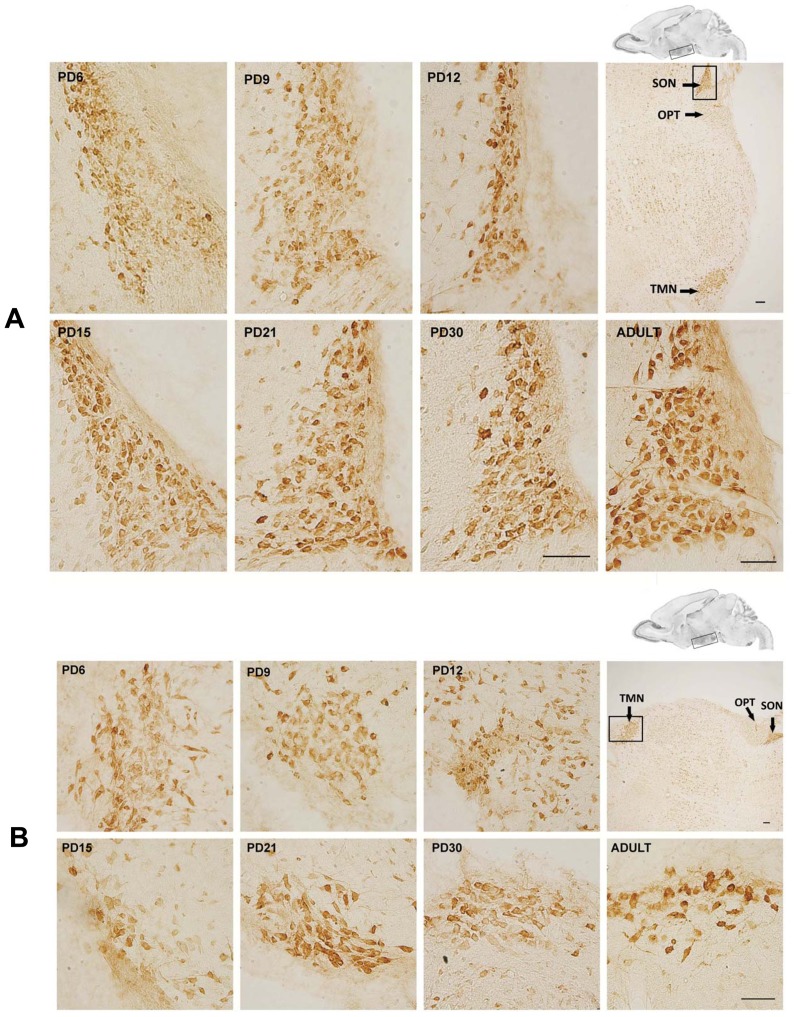
MANF expression in the hypothalamus. Rat pups were sacrificed at the indicated postnatal days. MANF expression in the hypothalamus was determined by IHC. IHC images were taken from the indicated area shown above. **A**: MANF expression in supraoptic nucleus (SON). **B**: MANF expression in tuberomammillary nucleus (TMN). OPT, optic tract. Scale bar = 100 µm.

MANF expression in the thalamus was wide spread. Interestingly, the anterodorsal thalamic nucleus (ADTN) displayed the highest MANF immunoreactivity in the thalamus (Fig. 6, indicated by arrows). Generally, the expression of MANF in the thalamus decreased during the postnatal neurodevelopment. For hypothalamus, the supraoptic nucleus (SON) and tuberomammillary nucleus (TMN) exhibited a particularly high level of MANF expression. Unlike other parts of brain, MANF expression in these two regions remained stable during the postnatal development and appeared a little higher in adult rats (Figs. 7A and B).

### MANF Expression in the Cerebellum

**Figure 8:**
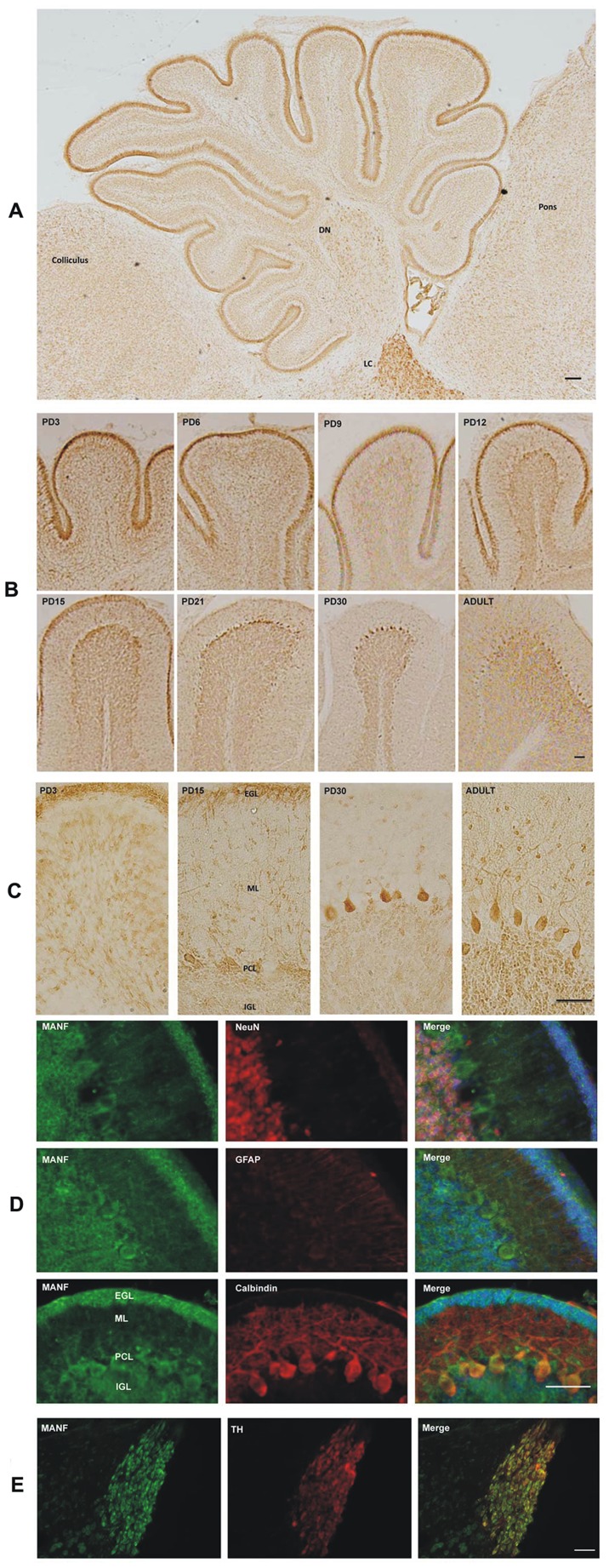
MANF expression in the cerebellum. **A**: MANF expression in the cerebellum of PD6 pups was determined by IHC. DN, deep cerebellar nuclei; LC, locus coeruleus. Scale bar = 100 µm. **B**: Rat pups were sacrificed at the indicated postnatal days. MANF expression in the cerebellum was determined by IHC. Scale bar = 100 µm. **C**: Images of higher magnification showing MANF expression in the external germinal layer (EGL), molecular layer (ML), Purkinje cells (PCs) and internal granule layer (IGL). Scale bar = 100 µm. **D**: Double-labeling immunofluorescent staining was performed to determine the identity of cells expressing MANF. The images showed the expression of MANF (green), NeuN (red), GFAP (red), Calbindin (red) and DAPI (blue) in the cerebellum of PD15 rat pups. Scale bar = 25 µm **E**: Co-localization of MANF (green) with tyrosine hydroxylase (TH) (red) in the LC of PD15 rat pups. Scale bar = 100 µm.

The cerebellum, a morphologically unique part of the brain, consists of folia which are separated by fissures. In the sagittal section of the cerebellum of rat pups of PD6, MANF immunoreactivity was observed in the external germinal layer (EGL), Purkinje cell layer (PCL), internal granule layer (IGL) and the deep cerebellar nuclei (DCN) (Figs. 8A and B). In early postnatal days (PD3 and PD6), strong MANF expression was detected in the EGL and DCN; its expression in these regions decreased as the brain developed (Figs. 8A and B; data on DCN were not shown). However, there was little change of MANF expression in the IGL during the development. Interestingly, MANF expression in Purkinje cells significantly increased from PD3 to PD30 (Fig. 8B, C). MANF was mainly expressed in neurons (NeuN-positive) in the IGL and calbindin-positive PCs (Fig. 8D). The cerebellar granule cell precursors (CGCPs) in the EGL also displayed strong MANF expression. These cells were NeuN-negative (Fig. 8D). Purkinje cells are NeuN-negative and calbindin-positive which is consistent with a previous report [20]. Very high MANF expression was shown in the locus coeruleus (LC) in the pons (Fig. 8A). The LC is composed of TH-positive neurons [21], [22]. Our results indicated that MANF was localized in TH-positive cells in the LC (Fig. 8E).

### MANF Expression in the Olfactory Bulb

**Figure 9:**
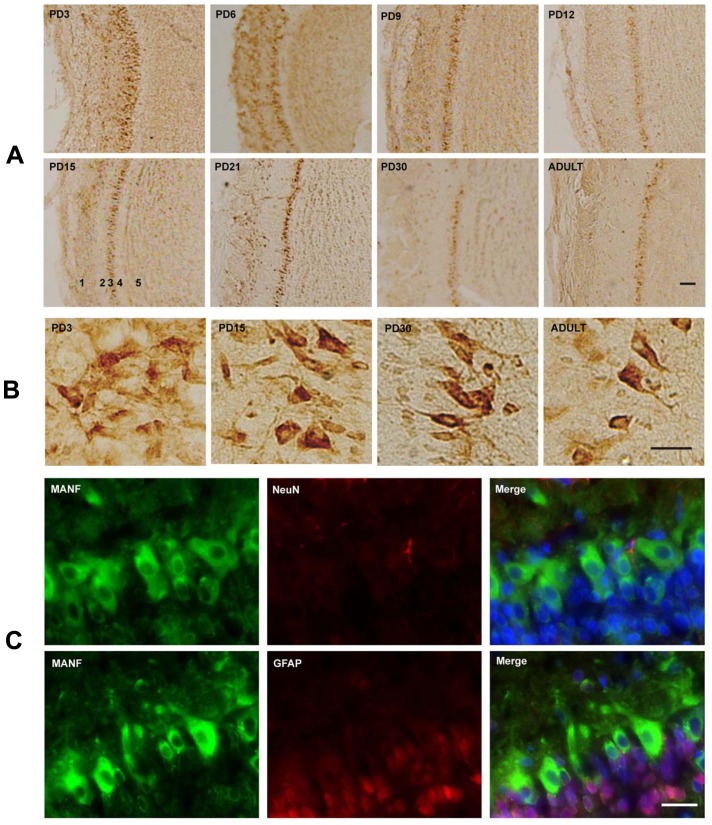
MANF expression in the olfactory bulb. **A**: Rat pups were sacrificed at the indicated postnatal days. MANF expression in the olfactory bulb was determined by IHC. 1, Glomerular layer (GLL); 2, External plexiform layer (EPL); 3, Mitral cell layer (MCL); 4, Internal plexiform layer (IPL); 5, Granule cell layer (GL). Scale bar = 100 µm. **B**: Images of higher magnification showed the MCL. Scale bar = 25 µm. **C**: Double-labeling immunofluorescent staining was performed to determine the identity of cells expressing MANF. The images showed the expression of MANF (green), NeuN (red), GFAP (red) and DAPI (blue) in the MCL of PD15 rat pups. Scale bar = 25 µm.

The main olfactory bulb has a multi-layered cellular architecture; they include glomerular layer, external plexiform layer, mitral cell layer, internal plexiform layer and granule cell layer. The highest level of MANF expression was detected in the mitral cell layer. Except for mitral cell layer, MANF expression in the olfactory bulb gradually decreased during the postnatal development (Fig. 9A). On PD3, mitral cell layer was a multi-layer structure; it became a mono-layer as the brain matured (Fig. 9B). Double-labeling immunofluorescent staining (Fig. 9C) indicated the cells in mitral cell layer were NeuN-negative as previously reported [20]. The mitral cells maintained a strong MANF expression during the postnatal development and the high expression persisted to adulthood (Fig. 9).

## Discussion

This study examines the spatiotemporal expression of MANF in the brain of postnatal and adult rats. High MANF expression is generally observed during the early PDs (PD3-6), then declines gradually as the brain matures. Early postnatal days are the period that critical developmental events such as neuronal migration, differentiation, synaptogenesis and neurogenesis, occur. High expression during this period suggests that MANF may be involved in these processes. Despite its name which is associated with astrocytes, our study indicates that MANF is predominantly localized in neurons in the CNS. This result is consistent with our previous findings in the adult rat brain, which show that MANF is mainly expressed in neurons but poorly expressed in glial cells [23]. Under focal cerebral ischemia, however, the expression of MANF was up-regulated in neurons as well as in astrocytes, oligodendrocytes and microglia [23]. This suggests that MANF may play dual roles in the CNS; it functions as a regulator of neuronal development and a neurotrophic/neuroprotective factor in response to insults. Under stress or pathological conditions, MANF may be induced from neurons as well as glia to alleviate the damages.

In the cerebral cortex, neurons in layer IV and VI display particularly strong MANF immunoreactivity. These layers consist of early-born neurons. Layer IV, the internal granular layer, contains different types of stellate and pyramidal neurons, and is the main target of thalamocortical afferents from thalamus type C neurons as well as intra-hemispheric cortico-cortical afferents [24]. Layer VI, the polymorphic or multiform layer, contains few large pyramidal neurons and many small spindle-like pyramidal and multiform neurons; layer VI sends efferent fibers to the thalamus, establishing a precise reciprocal interconnection between the cortex and the thalamus [25]. Our data suggest that MANF may be involved in the maturation of these neurons.

MANF is implicated in the development and survival of dopaminergic neurons [15]–[17]. We show here that the SNpc displays high MANF expression while the SNpr has a weak expression; this is consistent with the role of MANF in maintaining dopaminergic neurons. Although MANF is expressed in dopaminergic neurons (TH-positive), it is also expressed in other cells (TH-negative) in the SNpc, supporting the notion that MANF is not specific for dopaminergic neurons. The caudate nucleus (Cpu) of the striatum which is highly innervated by dopamine neurons from SNpc and the ventral tegmental area (VTA) also expresses MANF. Previous studies focused on the role of MANF in the development of dopaminergic neurons [15]–[17]. Since, MANF expression is not limited to dopaminergic neurons, the impact of MANF deletion on other neurons needs to be carefully evaluated.

Cornu Ammonis (CA) region of hippocampus is characterized by a thin layer of densely packed pyramidal cells. There are three main subdivisions: area CA1, CA2, and CA3. The CA field is bordered on one side by the dentate gyrus (DG); this is generally referred to as the proximal part (CA3). The opposite distal side is where CA1 meets the subiculum (SUB). In early postnatal days (PD3), strong MANF expression is observed in all CA regions (CA1-3) and subiculum (SUB). The granule cell layer (GCL) in the DG also expressed strong MANF. This layer contains granule cells that project to the CA3. In the thalamus, high expression is observed in the ADTA. The ADTA is part of the limbic system and plays an important role in the control of neuroendocrine and sympathetic-adrenal function [26]. In these structures, the expression of MANF declined as the brain matures.

In some structures, however, the expression of MANF remains high during the postnatal period and the high levels persist into adulthood. For example, the supraoptic nucleus (SON) and tuberomammillary nucleus (TMN) in the hypothalamus exhibit particularly high levels of MANF expression. These two nuclei consist of a cluster of magnocellular cells. The cell bodies in the SON produce a peptide hormone called anti-diuretic hormone while the neurons in TMN release histamine and is involved with the control of arousal, sleep and circadian rhythm. The expression of MANF in PCs of the cerebellum gradually increases from PD3 to 30 and maintains a high level during adulthood. The expression of MANF in the IGL of the cerebellum is stable throughout the postnatal development. The locus coeruleus (LC) in the pons is a norepinephrine (NE)-containing nucleus that modulates many physiological and pathological conditions including the sleep-waking cycle, movement disorders, mood alterations, convulsive seizures, and the effects of drugs such as psychostimulants and opioids. High expression of MANF is observed in the LC throughout the postnatal development; this warrants further study of MANF’s role in this nucleus. Mitral cells in the OB are the primary output cell from the olfactory bulb conveying olfactory sensory information to higher cortical areas. Consistent high expression of MANF is observed in mitral cells during the postnatal period and in adulthood. MANF is localized in the cell body of mitral cells. In summary, the expression MANF was developmentally regulated and mainly localized in neurons. MANF may play a role in the maturation of the CNS.
